# Hyperglycaemia Enhances Nitric Oxide Production in Diabetes: A Study from South Indian Patients

**DOI:** 10.1371/journal.pone.0125270

**Published:** 2015-04-20

**Authors:** Ramu Adela, Susheel Kumar Nethi, Pankaj K. Bagul, Ayan K. Barui, Saidulu Mattapally, Madhusudan Kuncha, Chitta R. Patra, P. Naveen Chander Reddy, Sanjay K. Banerjee

**Affiliations:** 1 Division of Medicinal Chemistry and Pharmacology, Indian Institute of Chemical Technology (CSIR-IICT), Hyderabad, 500007, India; 2 Cardiology Division, Mediciti Hospital, Hyderabad, 500063, India; 3 Biomaterials Group, Lipid Science and Technology Division, Indian Institute of Chemical Technology (CSIR-IICT), Hyderabad, 500007, India; University of California, Davis, UNITED STATES

## Abstract

**Background:**

We have previously reported that increased glucose levels were associated with higher serum nitric oxide (NO) levels in fructose-fed insulin resistant rats. However, the relationship between hyperglycemia and serum NO level was not clear. Therefore, the present study was designed to find the association between hyperglycemia and serum NO levels in Type 2 diabetic (T2DM) patients and T2DM with cardiovascular complication.

**Methods:**

Endothelial cells (HUVEC) were treated with of D-glucose (10-100mM), and NO levels and NOS gene expression was measured. Hyperglycaemia was induced in Sprague-Dawley rats, and serum NO levels were measured after 8 weeks. For clinical evaluation, five groups of patients were recruited: Control (CT, n=48), Type 2 diabetes (T2DM, n=26), T2DM with hypertension (DMHT, n=46), Coronary artery diseases (CAD, n=29) and T2DM with coronary artery diseases (DMCD, n=38). NO (nitrite + nitrate) levels were measured from human serum.

**Results:**

We found a significant (p<0.05) and dose-dependent increase in NO levels in HUVEC cells after 4 hours of high glucose exposure. eNOS and iNOS gene expression was increased in HUVEC cells after different concentrations and time periods of glucose treatment. We also observed significant (149.1±25μM, p<0.01) increase in serum NO levels in hyperglycaemic rats compared to control (76.6±13.2μM). Serum NO level was significantly higher in T2DM (111.8 μM (81.7-122.4), p<0.001) and DMCD patients ((129.4 μM (121.2-143.5), p <0.001) but not in CAD patients (76.4 μM (70.5-87)), as compared to control (68.2 μM (56.4-82.3)). We found significantly lower NO levels (83.5 μM (60.5-122.9)) in subjects suffering from diabetes since more than 5 years, compared to subjects (115.3 μM (75.2-127.1), p<0.001) with less than 5 years.

**Conclusion:**

In conclusion, high NO levels were observed in South Indian diabetic patients. Higher glucose levels in serum might be responsible for activation of endothelial cells to enhance NO levels.

## Introduction

Metabolic syndrome is a serious health concern. In United States, 35% of adults are at high risk of developing cardiovascular diseases, diabetes, stroke, atherosclerosis and coronary artery disease [1]. The global burden of diabetes mellitus has been estimated at 382 million and going to rise to 592 million by the year 2035 [2]. Number of Type 2 diabetes mellitus (T2DM) patients is increasing in both developed and developing countries but 80% contribution is from low and middle income countries [2]. Increasing incidence of morbidity and mortality due to cardiovascular complications including coronary artery diseases has been observed in Type 2 diabetic patients [3].

Diabetes is a metabolic disorder characterised by chronic hyperglycaemia. The long-term effects of diabetes mellitus include cellular injury, inflammation and failure of various organs [4]. The complications of diabetes are divided into macro vascular complications i.e., coronary artery diseases, peripheral vascular disease and stroke and, micro vascular complications i.e., diabetic nephropathy, retinopathy and neuropathy [5]. Among all complications, endothelial dysfunction is a common problem in all diabetic patients. Endothelial cells secrete different mediators such as vasodilators i.e., nitric oxide, and vasoconstrictors i.e., endothelin-1. Hyperglycaemia and other metabolic changes may lead to impairment of nitric oxide (NO) production [6]. Impairment of endothelial function in T2DM patients ultimately leads to cardiovascular diseases. Thus, endothelial dysfunction is the early feature of cardiovascular complications in T2DM [7].

Nitric oxide is a gaseous molecule secreted by the endothelium and a major modulator of endothelial function [8]. NO is synthesized from L-arginine by the family of enzymes called nitric oxide synthases (NOSs) viz. neuronal NOS (nNOS), endothelial NOS (eNOS) and inducible NOS (iNOS) [9]. NO is a key regulatory molecule with extensive metabolic, vascular, and cellular effects [10]. While low levels of NO is beneficial for several physiological and cellular functions, high levels of NO may cause detrimental effects in the cells. High levels of NO may react with superoxide anion to generate peroxynitrite radical, which binds to proteins and thus affects their function [11]. Altered serum NO levels in T2DM were reported by different investigators previously [12–14]. The serum NO data in T2DM patients that reported by different scientific literature is controversial. Some research articles reported increased NO levels in diabetes patients [13] whereas others reported the opposite [14]. In the present study, we have considered diabetic patients as a separate group compared to other diabetic patients with cardiovascular complications. We were interested in knowing if the serum NO levels were altered due to the duration of diabetes and the presence of any cardiovascular complications along with diabetes. Till now, there was no study that reported the nitric oxide levels in diabetic patients having cardiovascular complications.

Therefore, the present study was designed to understand the alteration in nitric oxide levels with T2DM and T2DM with cardiovascular complications in South Indian patients and to find whether hyperglycaemia can induce NO production in endothelial cells. Our study showed an increased level of nitric oxide in Indian T2DM patients, but not in patients with coronary artery disease (CAD) alone. Similarly, diabetic rats with hyperglycaemia also showed higher NO levels as compared to controls. Our study indicated that hyperglycaemia is responsible in generation of high levels of NO from HUVEC cells through induction of iNOS and eNOS gene expression.

## Materials and Methods

### Clinical study

#### Patient selection

Sample of 189 patients, including men and women aged 35–65 years, was taken from the Mediciti Hospital, Hyderabad, a city from southern part of India.

Subjects were allocated into five study groups mentioned below:

- Group 1: Control (CT) subjects (n = 50)
- Group 2: Type 2 diabetes mellitus (T2DM) subjects (n = 26)
- Group 3: Type 2 diabetes with hypertension (DMHT) subjects (n = 46)
- Group 4: Coronary artery disease (CAD) subjects (n = 29)
- Group 5: Coronary artery disease with Type 2 diabetes mellitus (DMCD) subjects (n = 38).

#### Consent process

The study conforms to the principles outlined in the Declaration of Helsinki and was approved by the Mediciti Ethics Committee (Institutional) Hyderabad. All patients were given detailed information of the study and they gave written consent before enrolling into the study.

#### Inclusion and exclusion criteria

**Group 1** Control (CT) subjects had no prior history of T2DM, hypertension, coronary artery diseases or any other cardiovascular diseases, and were not taking medication for any chronic medical condition. Fasting blood glucose, HbA1c and blood chemistry were normal. **Group 2** (T2DM) included subjects with HbA1c levels ≥ 6.5% as per American Diabetes Association (ADA) guidelines with proven history of T2DM but no other complications. **Group 3** (DMHT) included subjects with HbA1c levels ≥6.5% and systolic blood pressure ≥140 mm of Hg and diastolic blood pressure ≥90 mm of Hg, with history of T2DM as well as hypertension. **Group 4** (CAD) included subjects with narrowing or blockage of one or more epicardial coronary artery with greater than 25% stenosis shown in coronary angiography and diagnosed by cardiologists. This group had no prior history of T2DM. **Group 5** (DMCD) included subjects with coronary artery disease as defined for group 4 but patient had HbA1c levels ≥6.5% and prior history of T2DM. Exclusion criteria defined for this study were clinical or laboratory evidence of liver failure, renal failure (plasma creatinine levels > 1.5 mg/dl), Type 1 diabetes, cancer, thyroid disease and pregnancy.

#### Selection process for patients with coronary artery diseases

Coronary artery disease patients were identified in the Cardiac Catheterization Unit of Mediciti hospital. After coronary angiogram, all patients were evaluated by cardiologists in the inpatient setting. If patients were found to have any evidence of CAD, demographic, clinical, and angiographic data were collected from all such patients. Fasting sample were collected prior to the percutaneous coronary intervention or coronary artery bypass graft.

#### Sample collection process

Clinical history and complete physical examination including measurements of blood pressure, was collected and conducted for all the participants. Blood samples were collected by venipuncture after an overnight fast, using Becton Dickison Vacutainer Red colour coded Tubes and blood was allowed to clot by leaving at room temperature for 15 min. After centrifugation at 1500 g for 15 min at 4°C, serum was collected in 1.2 ml cryo vials and immediately stored at -80°C until testing.

#### Anthropometric measurement and biochemical parameters

Height and weight were obtained using standardised techniques and instruments. The body mass index (BMI) was calculated as the weight in kilograms divided by the square of the height in meters [weight (kg)/Height (m^2^)]. Fasting blood glucose levels were measured by the FreeStyle optimum glucometer (Abbott Diabetes Care, Australia). HbA1c levels were measured by the Bayer A1C Now^+^ Multi test A1C system (Catalog no.08842610). Creatinine (Siemens #DF33A) and uric acid (Siemens #BA4007) were measured by Siemens automated analyser (Dimension Xpand ^plus^), estimated Glomerular Filtration Rate (eGFR) calculated from creatinine by using Modification of Diet in Renal Disease (MDRD) formula. Serum Insulin and C-peptide levels were measured by Bio-Rad Multiplex assay kit (Catalog:#171-A7001M). Assay was performed as per the manufacturer instructions.

### Animal study

All animal experiments were undertaken with the approval of Institutional Animal Ethical Committee of Indian Institute of Chemical Technology (IICT), Hyderabad, India. Male Sprague-Dawley (SD) rats weighing between 180–220 gm were purchased from Teena Labs, Hyderabad, India. Animals were housed in BIOSAFE, an animal quarantine facility of IICT. The animal house was maintained at temperature 22 ± 2°C with relative humidity 40 ± 15% and 12 hour dark/light cycle throughout the study. Rats were randomly divided into two groups (n = 12). Rats were given a single dose streptozotocin (50mg/kg, i.p) in freshly prepared citrate buffer. After 72 hours of fasting, blood glucose level of rats was measured. Rats with blood glucose more than 200 mg/dl were included in the diabetic group (Dia group). Control group rats (Con group) were administered equal volume of citrate buffer (i.p) to nullify its effect. Rats were given free access to water and food throughout the study. After 8 weeks, blood was collected from rats to fractionise serum for further biochemical analysis.

#### Human and rat serum nitric oxide (NO) measurement

Detection of total nitric oxide (nitrite + nitrate) was performed with nitric oxide colorimetric assay kit (Arbor assay kit, MI, USA). In brief serum was deproteinized with 10% TCA (Trichloroacetic Acid) and the sample was centrifuged at 14,000 rpm for 20 min at 4°C to remove the precipitated protein. Resulting supernatant was used to measure the NO levels according to manufacturer’s instructions. The absorbance of the solution was read on a plate reader at 540 nm. To quantify the NO production, a standard curve was generated using sodium nitrite.

### Cell culture work

Human umbilical vein endothelial cells (HUVECs) were purchased from Lonza and cultured in endothelial basal medium (EBM; Lonza) supplemented with hydrocortisone, bovine brain extract, epidermal growth factor, ascorbic acid, gentamicin and 5% fetal bovine serum (FBS) in an incubator maintained at 37°C and 5% CO_2_ [15,16]. The cells of the passage three (P3) were used for performing the experiments.

#### Measurement of nitric oxide released from HUVEC cells

Human umbilical vein endothelial cells (HUVEC) were seeded in a 96 well plate at a density of around 1X 10^4^ cells per well and grown to confluence. To simulate the *in-vivo* diabetic conditions, the cells were incubated with 10, 25, 50 and 100 mM D-Glucose (Sigma) for two different time periods of 4 and 8 hours. We added different concentration of mannitol to each well during experimentation to maintain similar osmolarity. Vascular endothelial growth factor (VEGF) was used as a positive control while the untreated cells served as control. After incubation, the supernatant media of each of the treatment groups were collected separately and their nitric oxide levels were quantified using nitric oxide measurement kit (Arbor assay kit, MI, USA). The exact concentrations of NO, produced in response to treatment with different concentration of glucose were quantified from the NO standard curve.

#### DAF-2DA imaging of nitric oxide

The HUVEC cells were seeded in a 24 well plate at a density of around 60% confluence and cultured. The cells were exposed to 10, 25, 50 and 100 mM D-Glucose treatments for two different time periods of 4 and 8 hours. After the incubation, the cells were washed and incubated with 5μM DAF-2DA (Sigma) for 30 min. Then the cells were washed repeatedly and fluorescence images were captured (Scale bar = 50μm) using a Nikon fluorescence microscope [17].

#### RNA isolation, cDNA preparation and gene expression by real-time PCR

RNA was extracted from Human Umbilical Vein Endothelial Cells (HUVEC), using TRIzol reagent (Sigma) according to manufacturer’s instructions with minor modifications. Briefly, 5 × 10^6^ endothelial cells were incubated with 1 ml of TRIzol reagent for 5 minutes. The resulting cell lysates were then mixed with 0.2 ml of chloroform for 5 minutes at room temperature and centrifuged at 11,500 rpm for 15 minutes at 4°C. The upper aqueous layer was collected into RNase-free Eppendorf tubes and mixed with 0.5 ml of isopropanol for 10 minutes. Samples were then centrifuged at 11,500 rpm for 15 minutes at 4°C. The supernatant was aspirated, and the pellet was resuspended by vortexing in 75% ethanol in DEPC-treated water, this Samples were then air-dried, and RNA quantity was measured via nanodrop at 260nm. RNA integrity was also checked by 1% agarose gel electrophoresis. DNase treatment was further carried out to remove DNA contamination from isolated RNA. cDNA was prepared from isolated RNA (5 μg) using 1μl of reverse transcriptase (RT) enzyme, 1 μl dNTP mix (10 mM), 4 μl 5X reaction buffer and 10 picomole oligo dT and RNase free water. Total 10μl reaction mixture was denatured at 72°C for 3 min followed by sudden cooling for 10min and extension at 42°C for 60–90min. After cDNA preparation, real-time PCR was performed for gene expression analysis. All primers (eNOS, iNOS and GAPDH) were designed by Primer3 software (http://frodo.wi.mit.edu/) and synthesized commercially (Eurofin, India). Detailed sequences of all primers used in this study have been summarized in Table 1. Ten nanogram of cDNA were analysed on StepOnePlus (Applied Biosystem) using Absolute SYBR Green ROX PCR Master Mix (Takara) as described before [18]. Fold-change analysis was based on normalizing with GAPDH.

**Table 1:** Sequence of primers used for real-time PCR.

Gene	Forward primer	Reverse primer	Size(bp)	Annealing Temp(°C)
**eNOS**	ACCCTCACCGCTACAACAT	GCTCATTCTCCAGGTGCTTC	198	58
**iNOS**	ACAAGCCTACCCCTCCAG	TCCCGTCAGTTGGTAGGTTC	158	57
**GAPDH**	AGTAGAGGCAGGGATGATGTT	CTTTGGTATCGTGGAAGGACTC	133	57

### Statistical analysis

**Fig 1:**
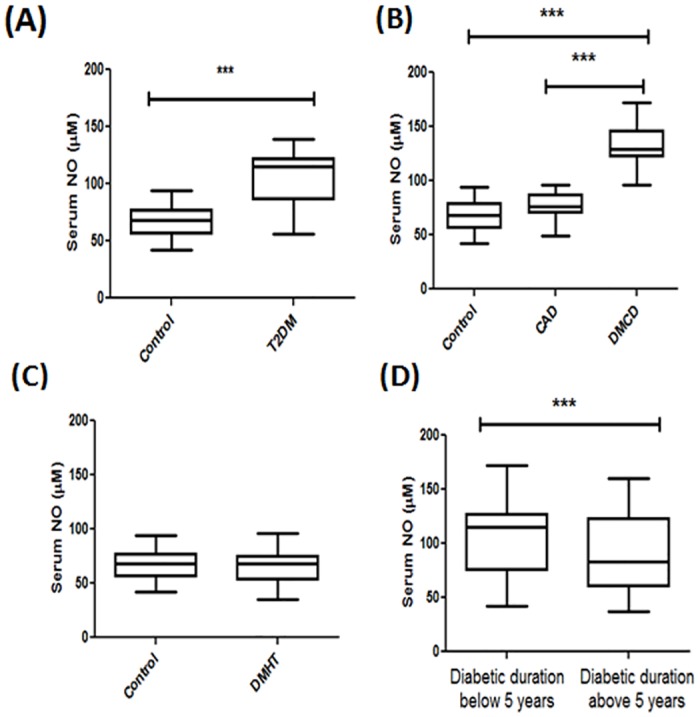
Serum nitric oxide levels in human subjects. A. Human serum NO levels in Control (CT) and Type 2 diabetes (T2DM). Data were represented as box (median (IQR)) and whisker plots. *** p<0.001 vs Control (CT). B. Human serum NO levels in Control (CT), Coronary artery disease (CAD) and Type 2 diabetes with coronary artery disease (DMCD). Data were represented as box (median (IQR)) and whisker plots. *** p<0.001 vs Control (CT), ^###^p<0.001 vs CAD. C. Human serum NO levels in Control (CT) and Type 2 diabetes with hypertension (DMHT). Data were represented as box (median (IQR)) and whisker plots. D. Human serum NO levels in two group of patients having diabetic duration below 5 years and above 5 years. Data were represented as box (median (IQR)) and whisker plots. ***p<0.001 vs diabetic duration below 5 years.

Patient clinical characteristics are represented as median (Inter Quartile Range (IQR)) for continuous and as percentages for categorical variables. Animal and cell culture results are expressed as mean ± SEM. Statistical comparisons were done by One way Analysis of Variance (ANOVA) for cell culture related results and t–test was used to see the difference between the two groups in animal study. The significance threshold (α) was set to p<0.05, unless otherwise mentioned. Non-normally distributed data is expressed as median (IQR). This was determined by using Kolmogorov-Smirnov test, followed by log transformation. Kruskal-Wallis test followed by Dunn’s multiple comparison analysis was used to find the significance in all the diseased groups with control; however the significance was presented separately in Fig 1A, 1B and 1C. Man-Whitney test was used to find clinical significance between NO levels in diabetics with disease diagnosed ≤5 years and >5 years. Spearman rank correlation was used to compare NO levels in fasting blood glucose (FBS) and glycated hemoglobin (HbA1c). ANOVA and Kruskal-Wallis and Man-Whitney, t-test analysis done by Graph Pad Prism version 5.01 and correlation analysis were performed by the SigmaPlot version 11.0.

## Results

### Description of patients

For this study, we took fifty healthy subjects that represent Group 1 (CT). While twenty six T2DM patients with no other complications were included in Group 2 (T2DM), forty six T2DM with hypertensive patients were included in Group 3 (DMHT). Total twenty nine coronary artery disease patients (twenty four acute myocardial infarction patients and five unstable angina pectoris patients) were included in the Group 4 (CAD). Total thirty eight T2DM with coronary artery disease patients (thirty one acute myocardial infarction and seven unstable angina pectoris patients) were recruited in Group 5 (DMCD). Single, double and triple vessel disease patients were included in both group 4 and group 5.

### Clinical characteristics of subjects

Table 2 shows the clinical characteristics of the study subjects. The mean age of T2DM, DMHT and CAD subjects were similar when compared to control subjects. However, the mean age of DMCD subjects was relatively higher when compared with controls. There was no significant difference in height, weight and BMI among all the groups. Systolic BP was similar in control and T2DM patients, but significantly higher in DMHT, CAD, and DMCD. Diastolic BP was similar in control, T2DM, CAD, DMCD patients, and higher in DMHT when compared to control and T2DM.

**Table 2:** Clinical and biochemical characteristics of the study subjects.

Clinical parameters	CT-Group 1 (n = 50)	T2DM-Group 2 (n = 26)	DMHT-Group 3 (n = 46)	CAD-Group 4 (n = 29)	DMCD-Group 5 (n = 38)	p value
N (Male/Female)	26/24	13/13	23/23	27/2	32/6	
Age (Years)	41.5 (38–50)	43 (40–54)	46 (40.2–53.7)	48 (43–55)	53 (45.5–56.5)^d^ **	p = 0.01
Height (Centimeters)	160.2 (154.3–167.3)	158 (154.3–164.5)	155 (150.5–162)	161 (154.3–165.0)	160 (152.1–164.5)	NS
Weight (Kg)	65.6 (57.6–75.7)	69.6 (59.9–75.2)	68.2 (60.6–75.9)	63 (55.6–68)	65.1 (58–73)	NS
BMI (Kg/cm^2^)	25.6(22.7–29.07)	25.5 (22.3–32.6)	27.9 (23.7–30.8)	24.9 (20–26.6)	25.7 (23.3–27.7)	NS
Systolic BP (mm Hg)	124.3 (116–131)	122 (111–130)	148.8(145–158)^b^ *** ^e^ ***	133.6(128–144)^h^ ***	133 (113–143.2)^i^ ***	p = 0.001
Diastolic BP (mm Hg)	79 (73.5–82.9)	76.6 (71–82)	93.1 (91.7–98.6)^b^ *** ^e^ ***	81 (76–88) ^h^ ***	82 (75.2–87) ^i^ ***	P = 0.001
Heart rate (beats per min)	79 (75.3–83.7)	80.5 (70–89)	87.6 (76–95.5)	72 (68–85)	85 (75.6–92.3)	NS
Fasting blood glucose(mg/dl)	97 (79–102)	177.5 (130–259.3)^a^ ***	188.5 (140–247.5)^b^ ***	96 (84–112) ^f^ *** ^h^ ***	192 (144–235)^d^ *** ^j^ ***	p = 0.001
HbA1c (%)	5.4 (5.2–6.1)	7.5(6.8–8.8)^a^ ***	8.3(7.5–9.7)^b^ ***	5.5 (5.2–5.6) ^f^ ***	8.5 (7.1–9.3)^d^ *** ^j^ ***	p = 0.001
Creatinine (mg/dl)	0.9 (0.7–1)	0.8 (0.6–1)	0.8 (0.7–1.1)	1 (0.8–1.1)	0.9 (0.8–1.2)	NS
eGFR	98.5 (85–117.8)	95.5 (80.2–113.5)	91 (81–99)	88.5 (73.7–105.3)	85 (69–108)	NS
Uric acid (mg/dl)	4.4 (2.2–5.7)	3.7 (3.1–4.5)	4.3 (3.4–5.2)	4.8 (4.3–5.7)	4.4 (3.4–5.6)	NS
Smoking History (Smoker/non smoker)	12/38	2/26	7/39	12/17	18/20	
Diabetic duration (Years)	_	2.6 (0.6–4)	4.0 (2–6)	_	4 (2–8)	
Hypertension duration (Years)	_	_	4.2(2–2.6)	3.4 (2–6)	4 (3–7)	
Glucose lowering therapy (%)	_	21(80.7%)	39(84.7%)	_	27(72%)	
Anti-hypertensive therapy (%)	_	_	35(76%)	11(37.9%)	19(50%)	

Results are expressed as Median (interquartile range).

^a^comparison between CT and T2DM,

^b^comparison between CT and DMHT,

^d^comparison between CT and DMCD,

^e^comparison between T2DM and DMHT,

^f^comparison between T2DM and CAD,

^h^comparison between DMHT and CAD,

^i^comparison between DMHT and DMCD,

^j^comparison between CAD and DMCD.

*p<0.05,

**p<0.01,

***p<0.001. NS-No significance.

### Biochemical characteristics of subjects

Table 2 shows the biochemical characteristics of the study subjects. T2DM, DMHT, DMCD patients had higher fasting blood glucose and HbA1c levels when compared to the control and CAD subjects. There was no significant difference in creatinine eGFR and uric acid levels among all the groups. Insulin and C-peptide levels were not significantly changed in the diseases groups as compared to control subjects, except diabetes with coronary artery diseases groups. (S1 Table).

### Duration and medical therapy among diabetic patients

The duration (median (Inter Quartile Range)) of diabetes among the subjects of three groups i.e., T2DM, DMHT and DMCD were (2.6 years (0.6–4)), (4.0 years (2–6)) and (4.0 years (2–8)) years, respectively. Similarly, the duration of hypertension among DMHT, CAD and DMCD subjects were (4.2 years (2.2–6)), (3.4 years (2–6)) and (4 years (3–7)) years, respectively. While 80.7% T2DM, 84.7% DMHT and 72% DMCD subjects were receiving glucose lowering therapy, 76% DMHT, 37.9% CAD and 50% DMCD subjects were receiving anti-hypertensive therapy. All CAD and DMCD subjects were receiving anti platelet therapy and other lipid lowering therapy for prophylaxis of the coronary artery disease.

### Serum nitric oxide levels in human subjects

Serum nitric oxide levels (median (Inter Quartile Range)) were significantly higher in T2DM (111.8 μM (81.7–122.4)) subjects as compared to control (68.2 μM (56.4–82.3), p<0.001) subjects (Fig 1A). There was no statistically significant difference in the NO levels between DMHT (68.2 μM (54.1–75.2)) and control (68.2 μM (56.4–82.3), p = 0.16) subjects (Fig 1B). Serum NO levels in DMCD (129.4 μM (121.2–143.5)) subjects were significantly higher when compared to control (68.2 μM (56.4–82.3), p<0.001) and CAD (76.4 μM (70.5–87), p<0.001) subjects. However there were no significant changes in CAD (76.4 μM (70.5–87)) subjects compared with control (68.2 μM (56.4–82.3), p = 0.072) subjects (Fig 1C).

All the diabetic subjects from T2DM, DMHT, DMCD groups were further divided into two groups based on duration of diabetes i.e., below 5 years (n = 69) and above 5 years (n = 41). Nitric oxide (83.5 μM (60.5–122.9)) levels were significantly low in subjects with diabetic duration >5 years when compared to subjects with diabetic duration <5 years (115.3 μM (75.2–127.1), p<0.001), (Fig 1D).

### Blood glucose and serum nitric oxide levels in streptozocin induced diabetic rats

**Fig 2:**
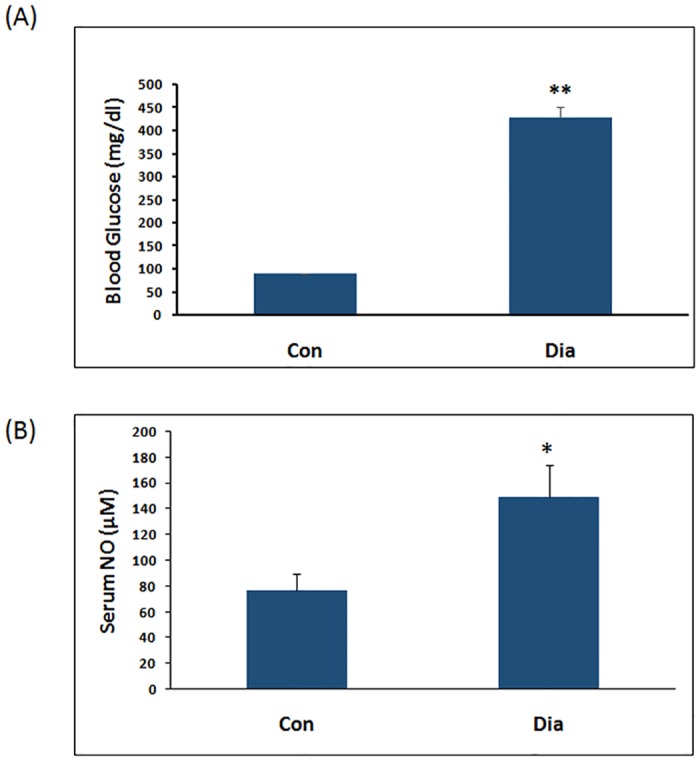
A. Blood glucose levels in diabetic rats (DIA) after 8 weeks of STZ administration. Data were represented as Mean ± SEM. (n = 8). **p<0.01 vs Control (CT). B. Serum NO levels in diabetic rats (DIA) after 8 weeks of STZ administration. Data were represented as mean ± SEM.*p<0.05 vs Control (CT).

As expected, blood glucose levels were significantly high in hyperglycaemic (Dia) rats induced by streptozocin (STZ), when compared to Control (Con) rats (Fig 2A). Serum nitric oxide levels (149.1±25 μM) in hyperglycaemic (Dia) rats were significantly high when compared to control (Con) rats (76.6±13.2 μM, p<0.01), (Fig 2B).

### Nitric oxide levels in HUVEC cells

**Fig 3:**
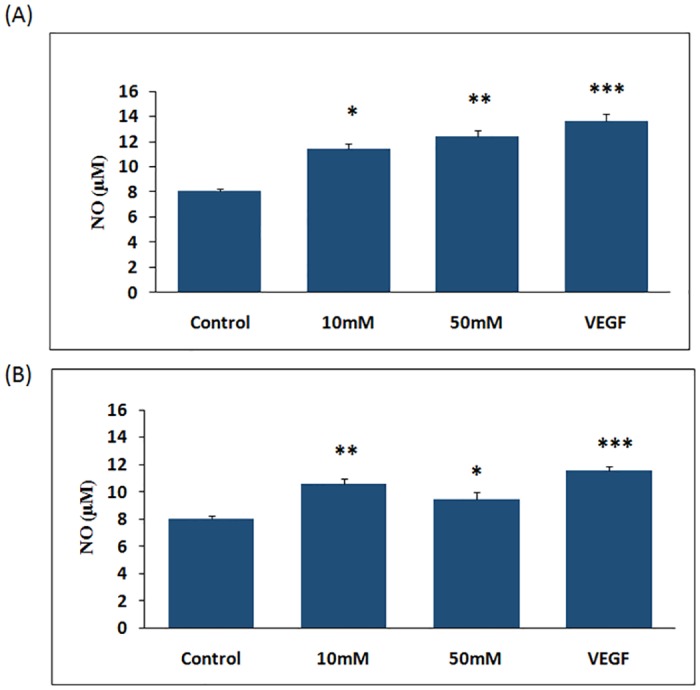
A. NO levels produced from HUVEC cells after 4hrs treatment of D-Glucose (10,50mM). Data were represented as ± SEM. * p<0.05, ** p<0.01 vs Control (CT). B. NO levels produced from HUVEC cells after 8hrs treatment of D-Glucose (10,50mM). Data were represented as mean ± SEM. * p<0.05, ** p<0.01, *** p< 0.001 vs Control (CT).

**Fig 4:**
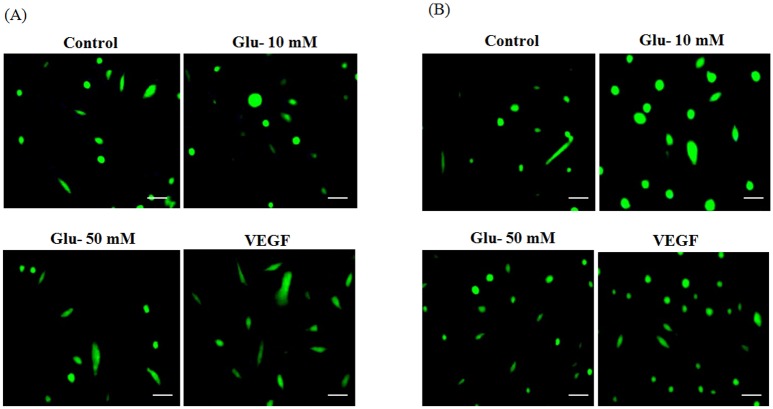
A. NO detection by fluorescent dye DAF 2 DA in HUVEC cells after treated with D-Glucose (10, 50mM) for 4 hrs. Scale bar = 50μm. B. NO detection by fluorescent dye DAF 2 DA in HUVEC cells after treated with D-Glucose (10, 50mM) for 8 hrs. Scale bar = 50μm.

We measured the NO levels in HUVEC cells in response to different concentration of D-glucose treatments (10, 25, 50 and 100mM) at different time points (4- and 8 hrs) (S1 and S2 Figs). Our data demonstrated dose-dependent increase in NO production in HUVEC cells after 4 hrs of glucose treatment (10 mM and 50 mM) (Fig 3A). Interestingly, this dose-dependent increase of NO production was not observed after 8 hrs of glucose treatment (Fig 3B). There is a decline in NO production in cells treated with 50 mM glucose treatment compared to 10 mM in 8 hours treatment time. To confirm these findings, we performed DAF-2DA imaging of NO in HUVEC. DAF-2DA (4, 5-diaminofluorescein diacetate) is a cell-permeable fluorescent molecule which directly measures the intracellular NO levels [19]. The fluorescence images are represented in the Fig 4 which illustrate a dose-dependent enhanced DAF-2DA fluorescence in HUVEC treated with 10mM and 50 mM D-Glucose concentration for 4 hours and 8 hours, respectively. For both the experiments, we treated the HUVEC cells with VEGF as standard to find the effect of hyperglycemia on NO production.

### iNOS and eNOS gene expression in HUVEC cells

**Fig 5:**
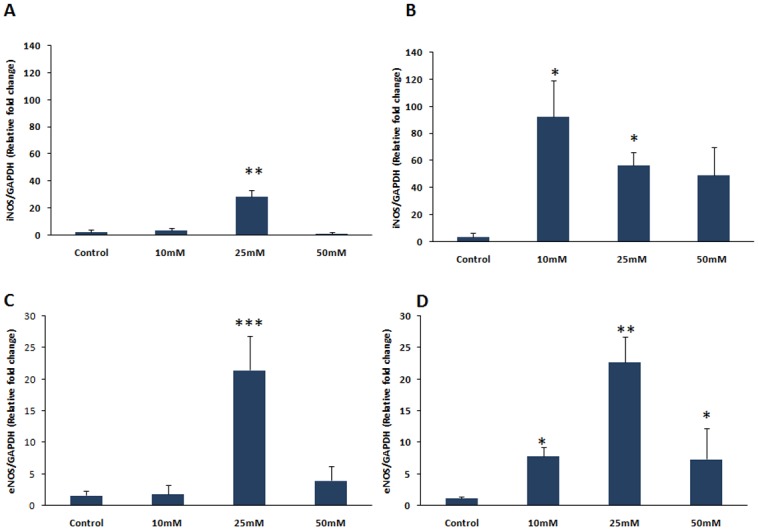
A. iNOS mRNA expression in HUVEC cells after 4hrs treatment of D-Glucose (10, 25, 50mM). Data were represented as mean ± SEM. **p<0.01 vs Control. B. iNOS mRNA expression in HUVEC cells after 8hrs treatment of D-Glucose (10, 25, 50mM). Data were represented as mean ± SEM.*p<0.05 vs Control. C. eNOS mRNA expression in HUVEC cells after 4hrs treatment of D-Glucose (10, 25, 50mM). Data were represented as mean ± SEM. ***p<0.001 vs Control. D. eNOS mRNA expression in HUVEC cells after 8hrs treatment of D-Glucose (10, 25, 50mM). Data were represented as mean ± SEM.* p<0.05,**p<0.01 vs Control.

To find out the mechanism of increased NO levels in HUVEC cells after the exposure to high glucose concentration, we measured iNOS and eNOS gene expression. Significant (p<0.05) increase in iNOS gene expression was observed in HUVEC cells after 4 hours (25mM) and 8 hours (10- and 25mM) of glucose exposure (Fig 5A and 5B). Similarly, eNOS gene expression was increased significantly (p<0.05) after 4 hours (25mM) and 8 hours (10, 25 and 50mM) of glucose exposure (Fig 5C and 5D).

### Correlation among NO, fasting blood glucose (FBG) and HbA1c in human samples

**Fig 6:**
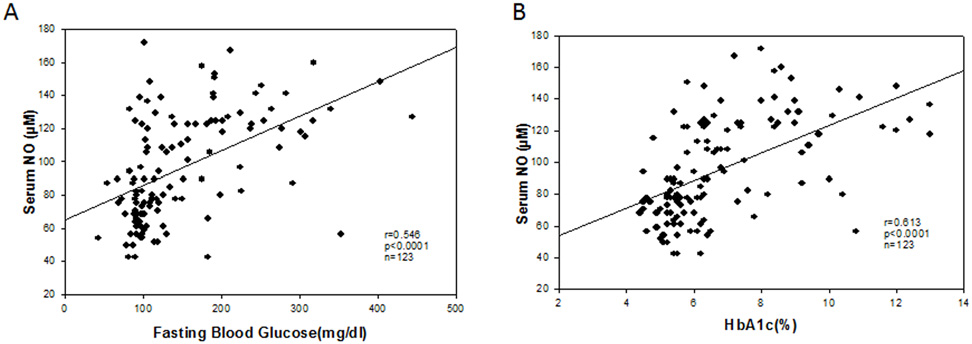
A. Scatterplot representing the correlation analysis of NO level and fasting blood glucose (FBG) (n = 123). A significant positive correlation exists between the NO level and FPG level (r = 0.543, p<0.0001) in all the groups except Type 2 diabetes with hypertension group. B. Scatterplot representing the correlation analysis of NO level and blood HbA1c level (n = 123). A significant positive correlation exists between the NO level and HbA1c level (r = 0.614, p<0.0001) in all the groups except Type 2 diabetes with hypertension group.

To find any correlation among NO, FBG, and HbA1c, we have done Spearman rank correlation coefficient analysis where we found a significant positive correlation between ‘NO and FBG’ (r = 0.543, p<0.0001, n = 123) (Fig 6A) and ‘NO and HbA1c’ (r = 0.614, p<0.0001, n = 123) (Fig 6B) in control, type 2 diabetes, coronary artery diseases and diabetes with coronary artery diseases. However, we have not included type 2 diabetes with hypertensive patients for this analysis. The plot is shown in scatter plots in Fig 6A and 6B respectively. Type 2 diabetes with hypertension patients NO levels were not correlated with the FBS (r = -0.0167, p<0.721, n = 43) and HbA1c levels (r = -0.322, p<0.0516 and n = 43).

## Discussion

In the present study, we investigated the alteration of serum nitric oxide levels in T2DM and CAD patients, and explored the correlation of higher nitric oxide levels with hyperglycemia. This is the first study to report and compare the NO levels among different groups of South Indian patients based on varying disease condition i.e., Type-2 diabetes, Type-2 diabetes with hypertension, Type-2 diabetes with coronary artery diseases and coronary artery disease. In our study, NO levels were significantly higher in Type-2 diabetic patients and Type-2 diabetic patients with coronary artery disease, when compared to both control and coronary artery disease patients. However, NO levels were not significantly increased in Type 2-diabetic patients in the presence of hypertension. Our data also indicate that levels of NO in blood of diabetic patients depend on the duration of diabetes.

According to previous literature, elevated levels of glucose may enhance NO production through increased expression of eNOS and iNOS gene and protein levels [20–23]. Increased levels of NO in *in-vivo* might have both beneficial as well as detrimental effect depending upon the NO concentration. On one hand, NO can cause relaxation of blood vessels and reduce hypertension, and on the other hand, NO may interact with superoxide radical (O_2_^−^) leading to inactivation of NO. The interaction of O_2_^−^ with NO is rapid and leads to the formation of potent oxidant radical, peroxynitrite. This may contribute to impaired endothelial function by stimulating arachidonic acid metabolism, lipid peroxidation, and prostanoid production [24, 25]. Our results clearly indicate that NO levels were increased in T2DM patients with coronary artery disease but not with hypertension. As per previous literature, plasma NO levels were increased in T2DM patients and not in non-diabetic patients with insulin resistance [26]. Our present data showing NO levels in Indian patients was consistent with previous study. Although several works showed increased levels of nitric oxide in T2DM patients [27], decreased levels were also reported by other investigators [28]. The conflict among the results of previous studies might be due to geographical location and genetic background of the population. Smoking and food habit may also affect serum NO levels as reported previously [29]. In the present study, diabetic subjects with 31.76% smokers (27 out of total 85 diabetic patients) showed higher NO levels as compared to control subjects with 31.57% smokers (12 out of total 38 total healthy patients). Despite having same percentage of smokers in both control and diabetic groups, we found significant difference in their serum NO levels. Therefore, our data ruled out the influence of smoking on alteration of serum NO levels among diabetic patients. Similarly, there was not any major difference in diet intake among all patients, as we have collected all patient samples from southern part of India. Our data also confirmed that there is no significant change in serum insulin, c-peptide and uric acid levels among control, diabetes and diabetes associated with hypertension. The reason for not finding any change in serum insulin and C-peptide levels in our study may be due to low number of patient samples. Previous study showed that insulin and C-peptide levels were increased significantly before 5 year of T2DM duration and then decreases with increasing the T2DM duration [30]. Moreover, in maturity-onset diabetes of the young (MODY) patients with low insulin responses, there are delayed and decreased insulin and C-peptide secretory responses to glucose due to beta cell dysfunction [31]. From the present study it is clear that serum NO level in diabetic patients was not influenced by above factors. However, diabetic complications and the stages of the disease may influence the serum NO levels in diabetic patients as observed by different studies. To confirm the correlation of diabetes with nitric oxide levels in patients, we collected diabetic patients both with and without cardiovascular complications i.e., hypertension and coronary artery disease (CAD). We found that increase in serum NO levels is positively correlated with FBG and HbA1C levels in all the groups of patients except type 2 diabetes with hypertensive patients.

In our study, we categorised all diabetic patients into two groups depending on the duration/progression of the disease. While some of the diabetic patients we recruited were in early stages of diabetes, others were late stage patients i.e more than 5 years of diabetes. To find out if duration of the disease affects the NO levels, we divided all the diabetic patients into two groups, early stage (less than 5 years) and late stage (more than 5 years). We observed significant alteration of serum NO levels in both groups. We found significantly lower NO levels in subjects having diabetes since more than 5 years compared to the subjects having a history of diabetes for less than 5 years. Our cell culture data with HUVEC cells also confirmed that high glucose exposure enhanced NO production at early time point but reduced subsequently after exposure for a longer duration.

Although we have observed increased nitric oxide levels in diabetic patients and diabetic patients with coronary artery diseases, there was no significant change in nitric oxide levels in diabetic patients with hypertension. Similarly there was no change in nitric oxide levels in patients with coronary artery disease alone. Our data indicate that the increased nitric oxide levels might be correlated with higher glucose levels in blood. However, increased glucose levels may not be able to increase nitric oxide levels when patients had concomitant hypertension. Several other studies also reported that reduced nitric oxide levels were observed in hypertensive patients as compared to control subjects [32–34]. However, in our study we have not observed any reduction of serum NO levels in diabetic with hypertensive patients. Therefore, it could be possible that hyperglycemia may influence to increase serum NO level in hypertension subjects to make a similar level as control. Previously, it was reported that nitric oxide levels were increased by the anti hypertensive therapy (angiotensin receptor blockers (losartan, olmesartan, telmisartan, valsartan) and angiotensin converting enzyme inhibitors (enalapril) [35–37]. In our study, 76% diabetic patients with hypertension were taking anti-hypertensive therapy (angiotensin receptor blockers, calcium channel blockers and beta blockers). However, anti hypertensive therapy could not able to influence on nitric oxide levels in diabetes with hypertensive patients. We believe that hypertension may impair endothelial function and reduce nitric oxide release. Previously, it has been reported that hypertension can produce structural damage to aortic endothelial cells in animals. Pressure overload due to hypertension is also associated with a direct toxic effect on human endothelium and impairment of the release of NO from vascular endothelial cells [38]. Similarly, Hoshiyama et al. showed that high glucose exposure increased eNOS protein expression, but decreased NO release in human glomerular endothelial cells. Decreased NO bioavailability was appeared to be associated with overproduction of superoxide and L-arginine deficiency [39]. All these changes may thus contribute to the reduced plasma NO concentrations in patients with essential hypertension. Decreased synthesis of NO might also result from abnormal handling of intracellular calcium and a consequent reduction in the activity of NOS [38]. Increased production of superoxide anions in oxidative stress, which rapidly deactivate NO, is a characteristic feature of experimental models of hypertension [40, 41]. It has also been shown that plasma indexes of lipid peroxidation are increased in patients with hypertension [42, 43]. We hypothesised that increased glucose levels in blood may enhance the nitric oxide levels in blood. However, it could be possible that other factors like geographical location, genetic background, smoking and food habit of the population or the progression of the disease may also be responsible for the change. To exclude the effect of all those parameters and prove that hyperglycaemia is solely responsible for the increased nitric oxide levels, we induced hyperglycemia in rats by giving streptozocin (STZ). Nitric oxide levels were measured in both the control and STZ treated rats. Similar to human study, increased hyperglycemia was associated with increased nitric oxide levels in STZ treated rats.

To explore the sources of nitric oxide production after hyperglycemia, we took human umbilical vein endothelial cells (HUVECs) and treated them with high concentrations of glucose. The main source of nitric oxide in blood is the endothelium. Hyperglycaemia in diabetic patients may alter endothelial function and cause vascular complications as well as dysfunction [44, 45]. Hence, in the present study we used HUVECs and treated them with 10–100mM D-glucose. Our *in-vitro* data showed that nitric oxide levels were increased in HUVEC cells after D-glucose treatment in a concentration-dependant manner. Altered gene and protein expression of NOS in endothelium cells after hyperglycaemia might be responsible for increased NO levels in blood. Although some literature [46, 47] showed decrease in eNOS activity after hyperglycemia, there are several other evidences which showed increase in eNOS gene and protein expression after high level of glucose exposure [20, 21]. It seems that the increased expression of eNOS depends on the cell type as well as glucose concentration and duration of exposure. Similarly, high expression of iNOS was also associated with hyperglycemia [22, 23]. Endothelial cells when treated with high glucose concentration can enhance iNOS expression [48, 49]. Similarly, our present study also demonstrated higher gene expression of both eNOS and iNOS in HUVEC cells after treating with high glucose concentration. However, their expression is dependent on the concentration and length of glucose exposure. Our data indicates that either iNOS or eNOS or both might be responsible for release of NO from endothelial cells. Although, we have not measured the iNOS and eNOS activity, most of the scientific literature showed that hyperglycemia mostly increased NO level through activation of iNOS [50–54]. In the present study, we have shown that endothelial cells are the source of NO production but the behavior of the endothelial cells differ among patients having “diabetes with hypertension” and “diabetes with coronary artery diseases”.

## Conclusion

In conclusion, higher NO level was associated with higher glucose level in diabetic South Indian patients both with and without coronary artery disease. There was no effect of insulin, C-peptide, uric acid and eGFR on increased serum NO levels in diabetic patients. *In-vitro* and animal studies confirmed that high glucose levels in serum might be responsible for enhanced nitric oxide levels in cells. Further work needs to be done to confirm whether higher nitric oxide levels in diabetic patients are beneficial or detrimental during the disease progression.

## Supporting Information

S1 Fig *A*] NO detection by fluorescent dye DAF 2 DA in HUVEC cells after treated with D-Glucose (10, 25, 50, 100mM) for 4 hrs (20X magnification). *B*] NO levels produced from HUVEC cells after 4hrs treatment of D-Glucose (10, 25, 50,100mM). Data were represented as ± SEM. * p<0.05,** p<0.01,*** p<0.001 vs Control (CT).
(DOCX)

S2 Fig *A*] NO detection by fluorescent dye DAF 2 DA in HUVEC cells after treated with D-Glucose (10, 25, 50, 100mM) for 8 hrs (20X magnification). *B*] NO levels produced from HUVEC cells after 8hrs treatment of D-Glucose (10, 25, 50, 100mM). Data were represented as mean ± SEM. * p<0.05, *** p< 0.001 vs Control (CT).
(DOCX)

S1 Table Serum Insulin and C-peptide levels in the study groups. (DOCX)
