# Supplementary material for: Hyperglycaemia Enhances Nitric Oxide Production in Diabetes: A Study from South Indian Patients
Source: PLoS One. 2015 Apr 20;10(4):e0125270. doi: 10.1371/journal.pone.0125270 (PMC4403926; doi:10.1371/journal.pone.0125270)
Supplement: S2 Fig — A] NO detection by fluorescent dye DAF 2 DA in HUVEC cells after treated with D-Glucose (10, 25, 50, 100mM) for 8 hrs (20X magnification). B] NO levels produced from HUVEC cells after 8hrs treatment of D-Glucose (10, 25, 50, 100mM). Data were represented as mean ± SEM. * p<0.05, *** p< 0.001 vs Control (CT). (DOCX) [file pone.0125270.s002.docx]

**S2.Fig.A**

**S2.Fig.B**

**S2.Fig: A]** NO detection by fluorescent dye DAF 2 DA in HUVEC cells after treated with D-Glucose (10, 25, 50, 100mM) for 8 hrs. Scale bar=50µm. **B]** NO levels produced from HUVEC cells after 8hrs treatment of D-Glucose (10, 25, 50, 100mM). Data were represented as mean ± SEM. * p<0.05,*** p< 0.001 vs Control (CT).
