# Supplementary material for: Hyperglycaemia Enhances Nitric Oxide Production in Diabetes: A Study from South Indian Patients
Source: PLoS One. 2015 Apr 20;10(4):e0125270. doi: 10.1371/journal.pone.0125270 (PMC4403926; doi:10.1371/journal.pone.0125270)
Supplement: S1 Table — (DOCX) [file pone.0125270.s003.docx]

**Table. S1.** Serum Insulin and C-peptide levels in the study groups.

|  | Control(n=22) | T2DM(n=20) | DMHT(n=24) | CAD(n=22) | DMCD(n=20) |
| --- | --- | --- | --- | --- | --- |
| Insulin (ng/ml) | 1.6 (0.4-3.1) | 1.6 (0.9-2.6) | 1.6 (1.1-2.4) | 2.5 (1.7-3.0) | 3.2 (1.9-4.1)* |
| C-peptide  (ng/ml) | 1.0 (0.4-1.9) | 1.2 (0.1-2.1) | 1.1 (0.8-1.6) | 1.0 (0.5-1.6) | 1.8 (1.3-2.8)* |

Data was expressed in median (IQR) *p<0.05 vs control subjects.
